# Clinical Benefits of a Randomized Allergy App Intervention in Grass Pollen Sufferers: A Controlled Trial

**DOI:** 10.1111/all.16558

**Published:** 2025-04-17

**Authors:** Caroline Holzmann, Johannes Karg, Matthias Reiger, Rajiv Kharbal, Paola Romano, Sabrina Scheiwein, Claudia Khalfi, Anna Muzalyova, Jens O. Brunner, Gertrud Hammel, Athanasios Damialis, Claudia Traidl‐Hoffmann, María P. Plaza, Stefanie Gilles

**Affiliations:** ^1^ Institute of Environmental Medicine and Integrative Health, Faculty of Medicine University Hospital Augsburg Augsburg Germany; ^2^ Institute of Environmental Medicine Helmholtz Munich—German Research Center for Environmental Health Augsburg Germany; ^3^ Outpatient Clinic for Environmental Medicine University Hospital Augsburg Augsburg Germany; ^4^ Institute for Digital Medicine University Hospital Augsburg Augsburg Germany; ^5^ Department of Technology, Management, and Economics Technical University of Denmark Copenhagen Denmark; ^6^ Next Generation Technology Region Zealand Denmark; ^7^ Health Care Operations/Health Information Management, Faculty of Business and Economics, Faculty of Medicine University of Augsburg Augsburg Germany; ^8^ Terrestrial Ecology and Climate Change, Department of Ecology, School of Biology, Faculty of Sciences Aristotle University of Thessaloniki Thessaloniki Greece; ^9^ Christine‐Kühne‐Center for Allergy Research and Education (CK‐CARE) Davos Switzerland

**Keywords:** allergic rhinitis, allergy app, clinical study, pollen forecast, symptom forecast

## Abstract

**Background:**

Symptom monitoring can improve adherence to daily medication. However, controlled clinical trials on multi‐modular allergy apps and their various functions have been difficult to implement. The objective of this study was to assess the clinical benefit of an allergy app with varying numbers of functions in reducing symptoms and improving quality of (QoL) life in grass pollen allergic individuals. The secondary objective was to develop a symptom forecast based on patient‐derived and environmental data.

**Methods:**

We performed a stratified, controlled intervention study (May–August 2023) with grass pollen allergic participants (*N* = 167) in Augsburg, Germany. Participants were divided into three groups, each receiving the same allergy app, but with increasing numbers of functions. Primary endpoint: rhinitis‐related QoL; Secondary endpoints: symptom scores, relevant behavior, self‐reported usefulness of the app, symptom forecast.

**Results:**

Rhinitis‐related QoL was increased after the intervention, with no statistical inter‐group differences. However, participants with access to the full app version, including a pollen forecast, took more medication and reported lower symptoms and social activity impairment than participants with access to a reduced‐function app. Using an XGBoost multiclass classification model, we achieved promising results for predicting nasal (accuracy: 0.79; F1‐score: 0.78) and ocular (accuracy: 0.82; F1‐score: 0.76) symptom levels and derived feature importance using SHAP as a guidance for future approaches.

**Conclusion:**

Our allergy app with its high‐performance pollen forecast, symptom diary, and general allergy‐related information provides a clinical benefit for allergy sufferers. Reliable symptom forecasts may be created given high‐quality and high‐resolution data.

## Graphical abstract

Different functions of an allergy app were tested for clinical benefit in a randomized, controlled trial in patients with grass pollen allergy. Participants with pollen forecast function took more medication and had fewer symptoms than participants without pollen forecast. A symptom forecast was developed based on the study data.

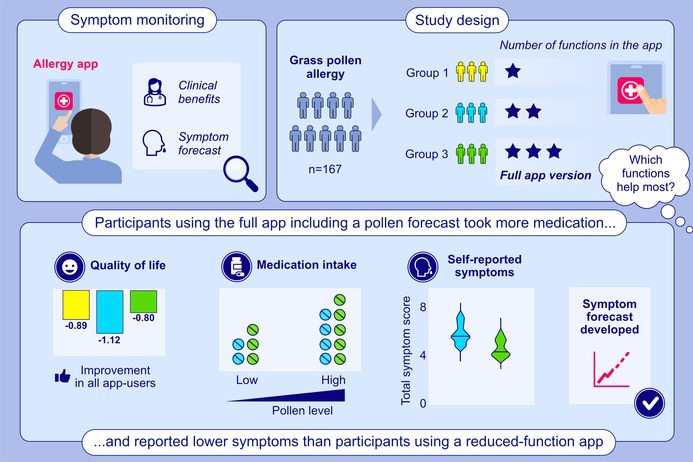

## 1 Introduction

In industrialized countries, airway allergies are among the most common noncommunicable diseases [1], causing high socioeconomic costs [2]. With the increasing relevance of allergies, which are also gaining importance in developing countries, interest in digital solutions such as allergy apps has grown [3]. Allergy apps could be a widely accessible and affordable solution for allergy self‐management [3].

In the rapidly evolving landscape of healthcare apps, more and more allergy apps are appearing on the market. Their purpose ranges from supporting the correct diagnosis, providing patient education, enhancing therapy adherence, monitoring side effects, and deriving personalized behavioral recommendations. Therefore, some allergy apps can be trained on the basis of user data. The features enable comprehensive patient engagement in the treatment process and improve long‐term symptom control through data‐driven insights [4]. Symptom monitoring has already been shown in a clinical study to improve adherence to daily medication [5]. However, the clinical benefit of an allergy app consisting of a symptom diary and an air quality forecast has never been demonstrated in a controlled trial.

The app function presumed to have the greatest significance for allergy sufferers is pollen forecasting, which can help to reduce exposure to allergenic pollen by providing timely warnings. In our pollen forecasting model, which was specially developed for the study, we integrated near real‐time data from automated pollen measurements and created an ensemble of seven machine learning models. This type of model is designed to improve accuracy and predictive power [6].

We developed a pollen forecast based on near real‐time pollen measurements, which we integrated into a new allergy app. With the app, we conducted the first stratified, controlled trial to investigate whether different features of the same app have a measurable impact on the quality of life (QoL), symptom severity, and exposure‐related behavior of pollen allergic individuals.

Using a large, well‐characterized group of participants and a long time series of symptom and behavior data, we examined the use of each app feature. Finally, a boosted decision tree model was used to predict the participants' symptoms based on patient baseline characteristics, symptom scores, and environmental data.

With our study, we aimed to fill a critical research gap in the area of digital health interventions for allergy sufferers. We hypothesized that the use of a complex allergy app featuring a pollen forecast and a symptom diary results in a significant reduction in symptom severity and an enhancement in QoL when compared to a control app with limited functionality. This approach is novel in the field, as allergy apps have, to our knowledge, never been assessed in such a controlled study design.

## 2 Methods

### 2.1 Participants Flow

**FIGURE 1:**
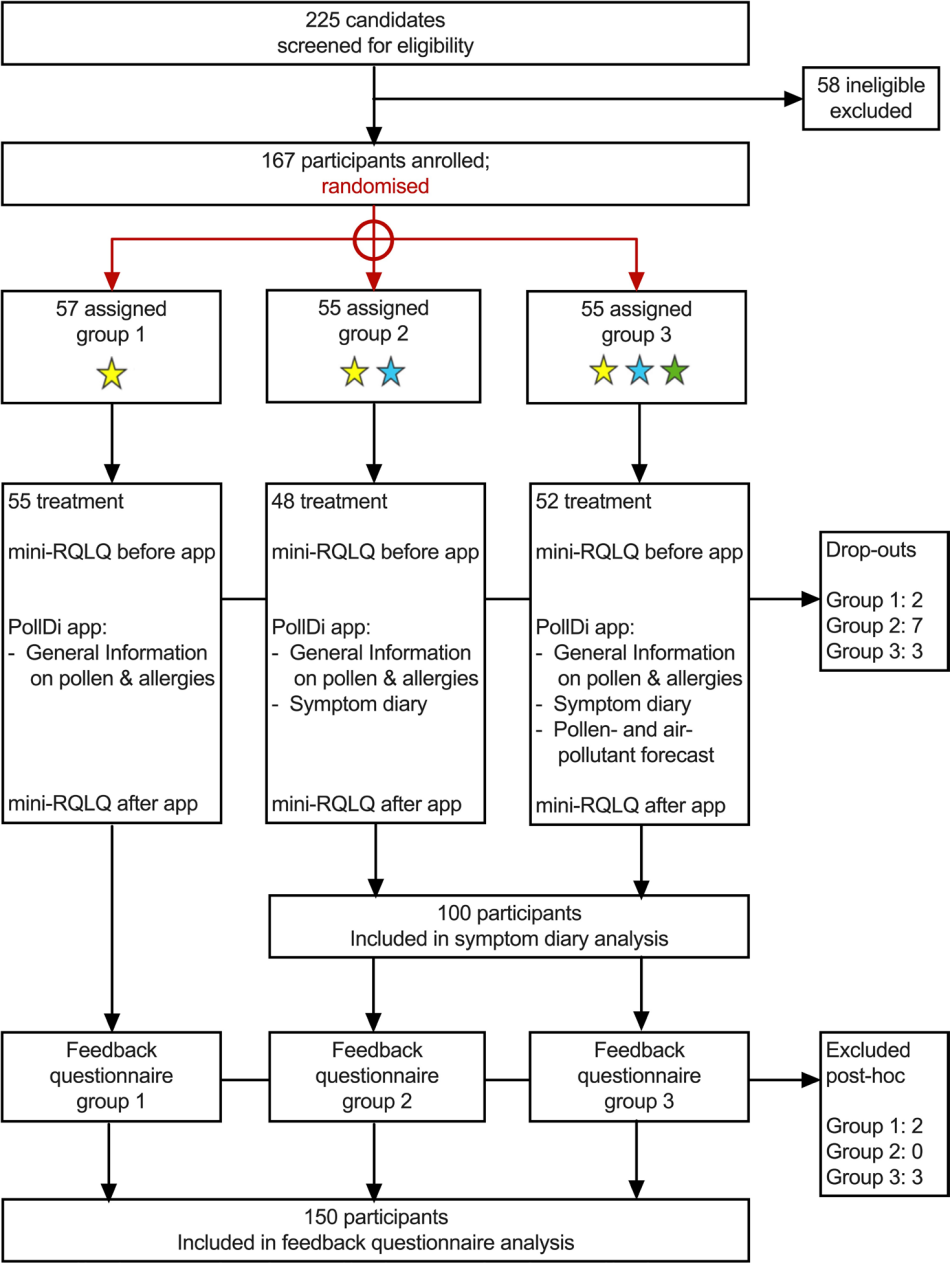
Participant flow.

The specifically developed PollDi app was tested for its clinical benefit in a stratified, controlled intervention trial with patients with grass pollen‐related seasonal allergic rhinitis (SAR; Figure 1). The study took place from May to August 2023 in Augsburg (+100 km radius), Germany. Participants were recruited among 255 eligible former study participants in the institute's database and de novo via flyers, posters, the institute website, and social media channels. Candidates underwent a two‐step eligibility screening. Based on calculations in G*Power, a total sample size of *n* = 175 was determined for the analysis of differences between the three study arms, assuming a medium effect size (*d* = 0.3), a power (*β*) level of 0.95, and an *α* level of 0.05. In total, 167 participants were recruited, leaving us eight participants short of the originally targeted sample size.

### 2.2 Inclusion and Exclusion Criteria

Inclusion criteria were being ≥ 18 years of age, the presence of allergic symptoms during the grass pollen season (rhinitis ± any other symptom; severity scale: 1–5), and sIgE (> 0.35 kU/L) against timothy grass pollen. Exclusion criteria were idiopathic or chronic rhinitis/sinusitis, systemic immunosuppressant therapy, severe illness, recent specific immunotherapy against grass pollen, perennial allergic symptoms, and absence > 4 weeks during the study period. Eligibility was first checked in a telephone interview, in which candidates were asked to report their average symptoms during a typical grass pollen season, both in terms of severity (0–5; 0 = no symptoms; 5 = very strong symptoms) and quality (rhinitis, conjunctivitis, asthma, atopic dermatitis, other symptoms), and to give information on confounding diseases and medication regimes. Eligible candidates were screened for grass pollen specific IgE by blood test (g6 ImmunoCAP, Phadia). Candidates who could prove a previous positive blood or skin prick test (result report no older than 10 years) were eligible without de novo testing.

### 2.3 Data Protection and Ethical Aspects

The data collected includes personal identifying information. The data was pseudonymized immediately after collection. The pseudonymized data was stored on a Veracrypt‐encrypted drive that could only be accessed by an authorized group of people. Written informed consent was obtained before the inclusion. The study was approved by the ethics committee of the Technical University of Munich (sign: 2022‐653‐S‐KH).

### 2.4 Randomization

The included participants (*N* = 167) were randomized into three intervention arms, with the randomization criteria age (≤ 35/> 35 years), sex (female/male), and average symptom severity (≤ 3/> 3) as reported in the screening. Masking: Randomization was carried out with a SAS script by two employees of the study center not involved otherwise in the study. All patient‐identifying personal and medical data were pseudonymized. For the app usage, each participant received a 12‐digit, group‐specific code (double‐pseudonym). Each group was identified by a number (1, 2, and 3). Of note, participants were aware of their group assignment based on the activated features of their app version. The patient flow is displayed in Figure 1.

### 2.5 Procedures

All participants daily used their specific version of the app (study website: https://www.poll‐di.de) every day, from 31 May to 31 August 2023. Analysis of the server data allowed the daily use of the application to be monitored. Individual missing usage data were treated as NA (not available) in the dataset. Participants with a gap of more than 4 weeks were excluded post hoc from the analysis.

For an illustration of the study design, see Figure S1. All groups received basic information and entertainment content via the app, covering the categories “myths and facts about pollen allergies,” “allergy quiz,” “questions & answers,” “ChatGPT gives answers on allergies,” and “fun facts.”

In addition, participants of Groups 2 and 3 received a symptom diary (see appendix of the Supporting Information) in their apps and were asked to daily document their symptoms of the previous day (for details, see Supporting Information). The diary had previously been used as a Qualtrics survey [7]; for the present study, it was implemented 1:1 in the PollDi app. The symptom‐related questions largely correspond to the “Pollen” app diary [8]. It included 40 questions on general well‐being, stress level, symptoms (eyes, nose, lungs), quality, and quantity of symptoms (on a scale of 0–3), medication use, and exposure‐related behaviors.

Only Group 3 received an additional short‐term pollen‐ and air‐pollutant forecast. The pollen forecasting model was trained on 6 years (2017–2022) of grass pollen data from Augsburg, measured by the BAA500 automated monitor (“PoMo”; Hund, Wetzlar, Germany). Data input were past daily pollen data (pollen/m^3^) and past and forecasted daily weather data (German Weather Service, DWD). An ensemble model calculated the pollen forecast for the present day and the following 2 days. It was provided as pollen levels based on literature [9, 10] and own unpublished results. For details on pollen forecast, see Supporting Information and Tables S1–S3.

The air pollutant forecast was based on a 7‐day moving average of daily air pollutants (Bavarian State Office for the Environment): fine particulate matter (PM_10_), nitrogen dioxide (NO_2_), and ozone (O_3_).

### 2.6 Primary Outcome

#### 2.6.1 Rhinitis‐Related QoL Impairment

A disease‐specific QoL index was calculated in a before‐and‐after survey using the standardized and validated miniRQLQ [11]. The questionnaire was filled in electronically via Qualtrics at the start (23 May–13 July 2023) and immediately after the intervention period (September 1, 2023). Participants of all three groups completed the mini‐RQLQ questionnaire. Results are controlled for confounders (study inclusion date, pollen exposure at inclusion, age, gender, mean symptom severity according to screening) and stratified.

### 2.7 Secondary Outcomes

#### 2.7.1 Symptom Scores

From symptom diary data, a Total Symptom Score (TSS) was calculated based on validated methods [12]. The TSS combined the general health rating, recorded on a scale of 0 to 10 (reciprocal value), and the severity of nasal, ocular, and bronchopulmonary symptoms, each rated on a scale of 0–3. A maximum TSS value of 0 to 19 could be achieved. We also calculated specific validated scores for each organ (nose, eye, lung), such as the Total Nasal Symptom Score (TNSS). This takes into account the severity of the nasal symptoms (0–3) and the quality of the nasal symptoms. These include itching, sneezing, runny nose, and nasal congestion. Furthermore, we determined the Total Nasal Symptom and Medication Score (TNSMS), which combines the TNSS and medication [12]. Medication was weighted in the calculation: 1.0 point for the use of “nose drops” or “tablets” (or both), 0.25 points for the use of “eye drops,” 0.5 points for “other” medication and 0.3 points for the use of “homeopathic” remedies.

Symptom scores could only be calculated for participants of Groups 2 and 3, since Group 1 had no access to the symptom diary (control group with “placebo” app).

For the symptom forecast, we categorized the recorded nasal, ocular, and pulmonary symptom severity scores in three classes (“no symptoms”, “mild symptoms,” and “moderate to strong symptoms”). Moderate and strong symptoms were combined to one single class to reduce the imbalance in symptom class frequencies.

#### 2.7.2 Feedback Questionnaire

A specifically developed feedback questionnaire consisting of up to 60 questions was used to assess the optical appeal and usability of the app and its different functions, as well as the subjective usefulness of the app in terms of symptom relief and QoL impact. The participants were only asked to evaluate aspects and functions of the specific app version they had received, that is, the three groups each received tailored feedback questionnaires (see appendix of the Supporting Information).

### 2.8 Statistical Methods

Statistical analyses were performed using R studio, version 4.3.1, GraphPad Prism, version 10.0.2 (232), and Python, version 3.11.5. Continuous data are presented as means (95% confidence intervals), while categorical data are reported as frequencies and percentages. A normality test was performed with the Kolmogorov–Smirnov method. All statistical tests were conducted with a significance level of *α* = 0.05. For the comparison of symptom scores between groups, Mann–Whitney tests were used. For the relationships of symptom scores with pollen concentrations, symptom scores were (ln)‐transformed to achieve a normal distribution. To examine co‐factor effects, two‐factor ANOVA mixed models with post hoc Dunnett's multiple comparisons test were used.

In the miniRQLQ before‐survey, missing values were imputed using a tensor‐based method [13, 14]. Therefore, the distribution of missing data was first analyzed by intra‐ and inter‐item correlations and identified as missing not at random (MNAR) data. The imputation method was evaluated by analyzing the variances before and after imputation, and scaling factors were used to calculate further group statistics. The CANDECOMP/PARAFAC (R package: rTensor) was used for tensor decomposition.

To forecast pollen, an ensemble of models was used that comprised sub‐models with varying configurations and weights, aggregating predictions from individual models to minimize the forecasting uncertainty. Seven sub‐models were selected for the final ensemble based on individual accuracy metrics [15] (Table S1): General Linear Model (GLM), Extreme Gradient Boosting (XGBoost), Neural Network timeseries (NNAR), Random Forest (RF), Support Vector Machine (SVM), Hybrid Prophet‐XGBoost, and autoregressive moving average (ARIMA). The development of the pollen forecast, the ensemble performance (Table S2) and its output data (Table S3) are described in detail in the Supporting Information.

The symptom forecast was developed using an XGBoost (Extreme Gradient Boosting) model, which is a state‐of‐the‐art machine‐learning algorithm based on gradient‐boosted decision trees that can handle both regression and classification tasks [16]. Categories of the nasal, ocular, and pulmonary symptom severity served as the target variable. Input features were patient‐derived baseline data (age, sex, screening data), environmental data, and data obtained in the symptom diary, such as symptom severity, symptom qualities, and behavior. Regarding the symptom severity and grass pollen concentration, three daily lags were included as input features. For the training of the models, 75% of the patients (*n* = 61) were randomly assigned to the training set and the remaining 25% of patients (*n* = 21) to the test set to avoid patient overlap. The applied hyperparameter for tuning of the model was:

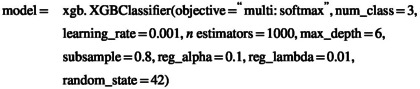




After training the model, the performance was evaluated on the test set using the metrics accuracy, precision, recall, and F1‐score as well as confusion matrices. To gain insights into the models' decisions, we further created SHAP (SHapley Additive exPlanations) summary plots to derive feature importance [17].

## 3 Results

### 3.1 Randomization

From 2 January to 15 July 2023, 225 candidates were screened for eligibility by telephone interview. 58 candidates were excluded due to inadequate sensitization, reported perennial symptoms, failure to be re‐contacted, or > 4 weeks planned absence during the study period. The remaining 167 candidates were randomized into the three study arms (Table 1). Twelve of them dropped out during the intervention. Five further participants did not return their feedback questionnaire and were therefore excluded from the respective analysis only.

**TABLE 1:** Baseline characteristics of the intention‐to‐treat population.

	Group 1	Group 2	Group 2
Sex			
Male	33.3%	30.9%	34.5%
Female	66.7%	69.1%	65.5%
Age (years)	31	32	35
Average symptom severity	3	3	3

Randomization (Figure S2) resulted in an equal distribution of the target variables between the study arms (Figure S3).

### 3.2 High‐Perfor‐mance Pollen Forecast

**FIGURE 2:**
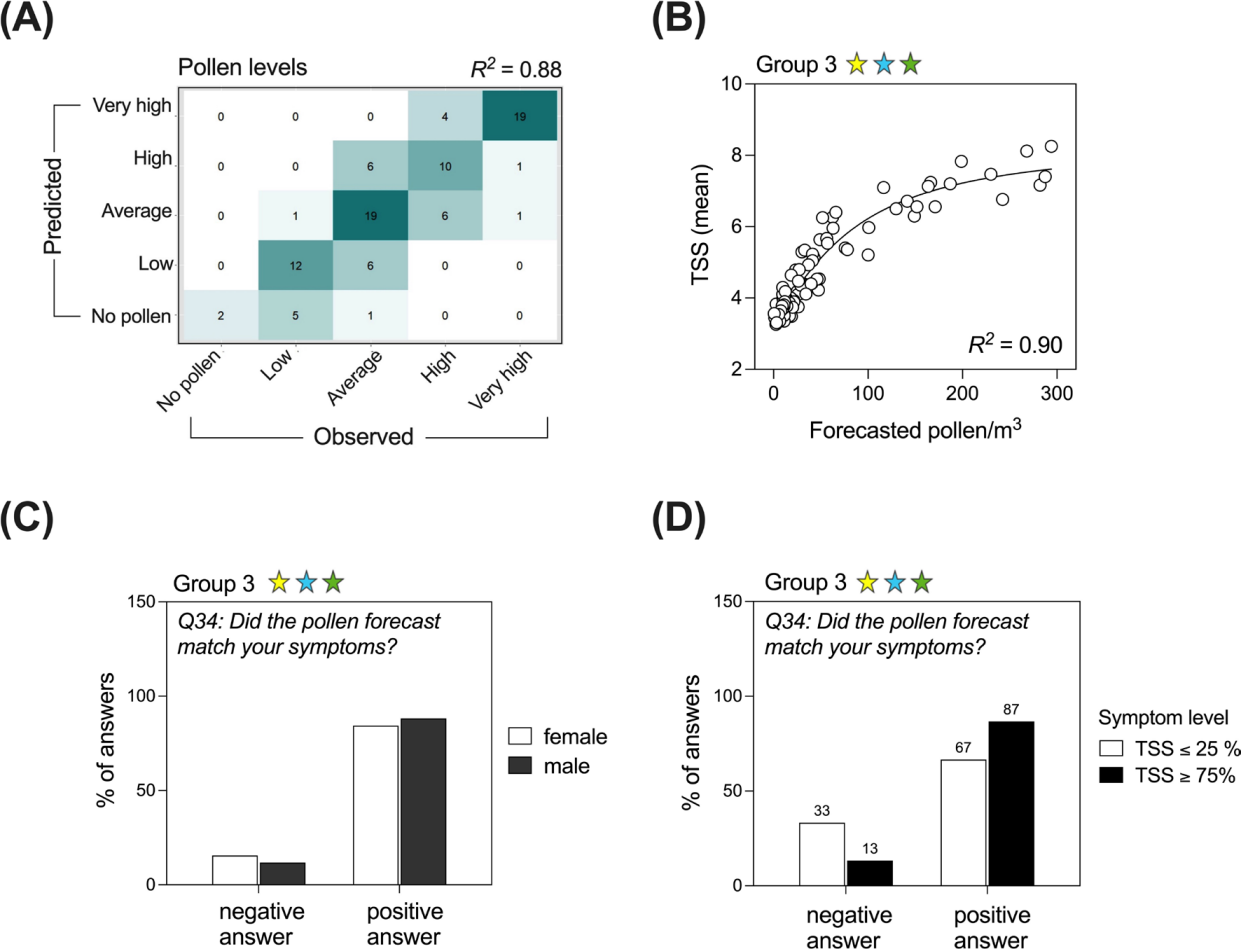
The pollen forecast and its relationship with symptom scores and perception of the participants. (A) Performance of the pollen forecast during the study period. (B) Correlation between the total symptom score of Group 3 and the forecasted pollen levels (line: 4‐parameter fit). (C, D) Accordance between pollen forecast and perceived symptoms of Group 3 participants, by gender (C) and by symptom level (D). TSS = total symptom score.

A pollen forecast was available only for Group 3 participants. The pollen forecasting model had a strong performance during the study period, with a tendency to overestimate low to medium pollen levels (*R* ^2^ = 0.88). The sensitivity was 0.61, the specificity 0.91, and the positive predictive value 0.73 (Figure 2A). In Group 3 participants, the TSS was significantly and positively correlated with the forecasted pollen concentrations (*R* ^2^ = 0.90; *p* < 0.0001; Figure 2B). To assess the individual experience of the participants, 150 completed feedback questionnaires were available (53 in Group 1, 48 in Group 2, and 49 in Group 3). There were 2 missing questionnaires in Group 1; 0 missing questionnaires in Group 2; and 3 missing questionnaires in Group 3. The question Q34 “*Did the app's pollen forecast match the severity of your actual symptoms?*” was answered with an affirming statement (“partially,” “strongly,” or “very strongly”) by most of the participants (female: 84.4%; male: 88.2%; Figure 2C). Of the participants whose TSS during the study period was ≥ 75th percentile (*N* = 15; average TSS = 6.4), 87% reported that the pollen forecast was consistent or very consistent with their symptoms. Of the participants whose TSS was lower than the 25% percentile (*N* = 12; average TSS = 1.5), 67% stated that their symptoms agreed with the pollen forecast (Figure 2D). A detailed overview over the answers to Q34, stratified by sex and TSS, can be seen in Table S4.

### 3.3 Reduced QoL Impairment Through Intervention

The first miniRQLQ survey had missing responses. Group 1 had 51 questionnaires with 6.3% missing responses, Group 2 had 44 questionnaires with 9.7% missing responses, and Group 3 had 51 questionnaires with 9.1% missing responses. In total, 8.4% of responses were missing in the pre‐intervention survey. As the data were not missing at random (MNAR) (chi‐squared test *p* < 2.2 × 10^−16^) and correlations existed in the data set, the missing data could be imputed without leading to imbalances in the important randomization criterion mean symptom severity (Figure S4).

**FIGURE 3:**
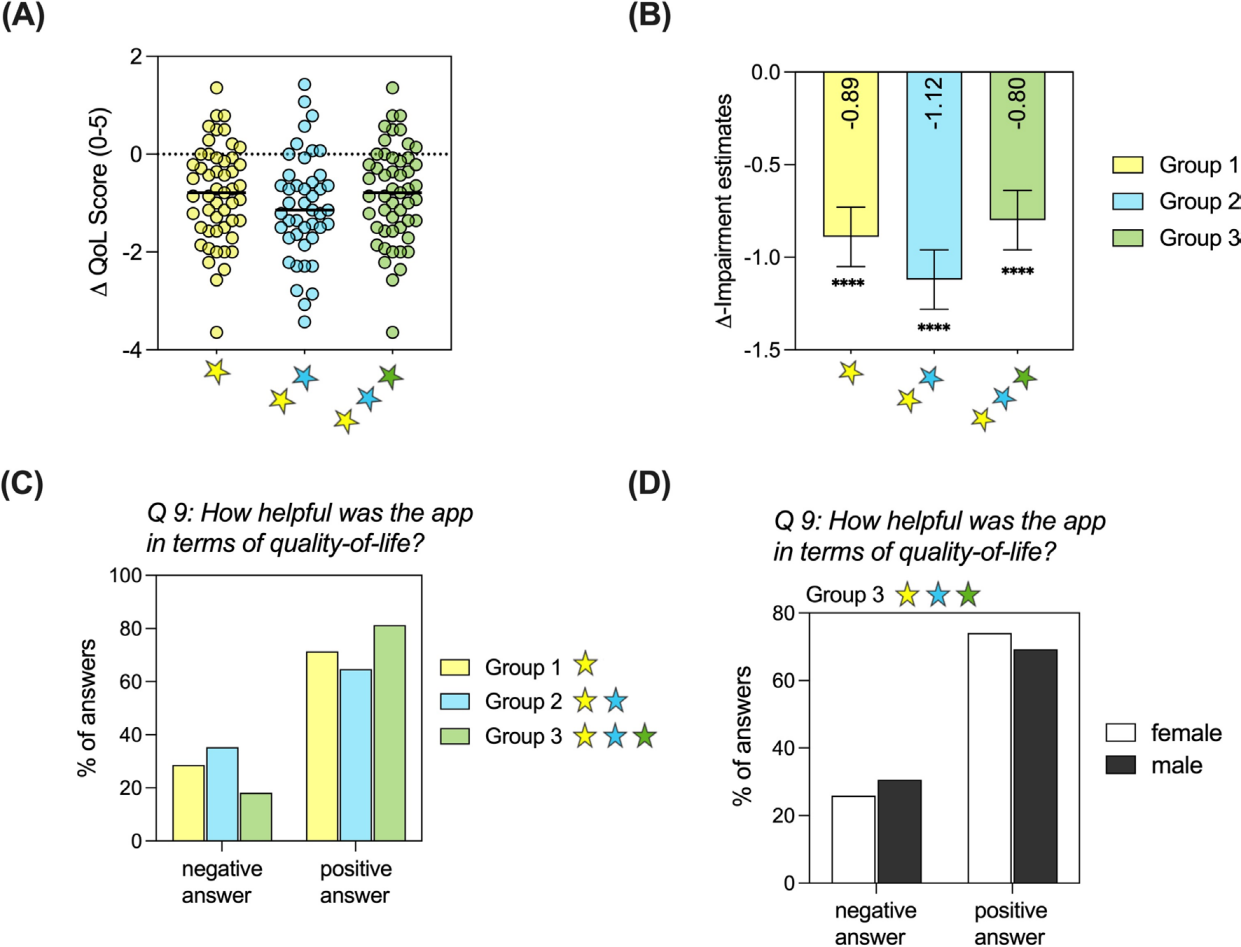
Impact of app usage on rhinitis‐related quality of life (QoL). The rhinitis‐related QoL was assessed before and after the intervention by miniRQLQ. Raw data (A) and GLMM (B) show a significant QoL improvement after versus before the intervention in all three groups, the inter‐group differences being not statistically significant. The usefulness of the app in terms of QoL was also assessed in the feedback questionnaire and was answered with a positive answer by the majority of participants, with no differences between groups (C) or sexes (D).

The QoL was significantly improved in all three groups after app use (*p* < 0.0001), with no significant differences between the groups (Figure 3A). Group 3 showed the smallest Δ QoL (median: −0.72; estimated Δ impairment: −0.80) compared to Group 1 (median: −0.84; estimated Δ impairment: −0.89) and Group 2 (median: −1.07; estimated Δ impairment: −1.12) (Figure 3B).

In addition to the miniRQLQ, QoL‐related items were also contained in the feedback questionnaire. After the intervention, the participants of all groups were asked (Q9): “*How helpful was the app in terms of quality of life?*” 81.6% participants of Group 3, 68.9% of Group 2, and 71.7% of Group 1 rated the app useful in terms of QoL (Figure 3C), with no significant differences between the groups (*p* = 0.18, Kruskal–Wallis test). In the sex comparison, 76/101 women (75.2%) and 34/49 men (69.4%) rated the app “somewhat helpful,” “helpful,” or “very helpful” (Figure 3D). A detailed overview over the answers to the question Q9, stratified by study group and sex, can be seen in Table S5.

An increased negative health awareness due to the app usage was negated by the majority of participants, with no significant differences between the three groups (Figure S5).

### 3.4 Fewer Symptoms and Social Activity Impairment and More Use of Medication With Access to Pollen Forecast

**FIGURE 4:**
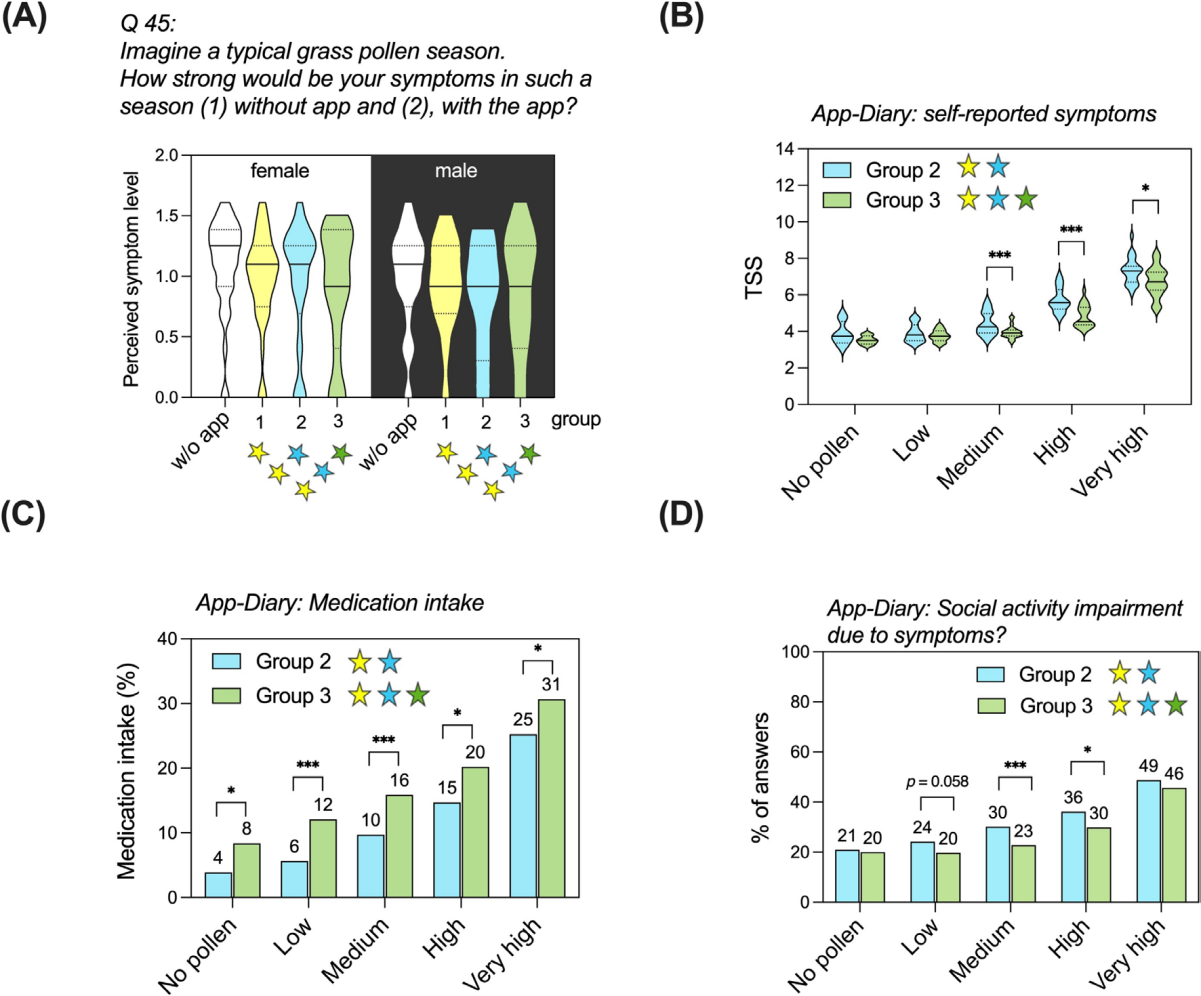
Comparison of symptoms, medication intake and social life‐impairment between groups. (A) Perceived symptom level in a typical grass pollen season with and without app, as indicated in the feedback questionnaire. Results are stratified by sex. (B) Symptom scores as per symptom diary of Groups 2 and 3, stratified by pollen level. *P < 0.05; ***P < 0.001 (two‐way ANOVA with post hoc Dunnett's test). TSS = total symptom score. (C) Medication intake of Groups 2 and 3, stratified by pollen level. *P < 0.05; ***P < 0.001 ($\chi_{\text{Pearson}}^{2}$). (D) Impairment in everyday social activities, stratified by pollen level. The distribution of answers differed significantly between Groups 2 and 3. *P < 0.05; ***P < 0.001 ($\chi_{\text{Pearson}}^{2}$).

When asked in the feedback questionnaire (Q45): “*Please indicate the level of symptoms during an allergy season, (1) without app usage, and (2) with app usage*,” the participants of all groups rated their symptoms lower with app than typically without app. Only in Group 3, symptoms were rated significantly (*p* < 0.0001) weaker with app usage than without. In Groups 1 and 2, the difference was not significant (Group 1: *p* = 0.59; Group 2: *p* = 0.11). The answers were not significantly determined by sex (*p* = 0.71), age (0.25), or a combined effect of sex and age (Figure 4A and Table S6).

We further investigated whether there was a measurable difference in outcomes, as recorded in the app's diary, between the Groups 2 (“semi‐full” app) and 3 (“full” app). The median time‐series length of diary entries was 86.5 days (average: 79.2; minimum: 13; maximum: 94 days); in Group 3, the median length the of time‐series was 82 days (average: 76.3; minimum: 14; maximum: 94 days). At medium and high pollen levels (≥ 10 pollen grains/m^3^), which occurred on 85% (80/94) days during the study period, Group 3 reported significantly lower overall symptoms (TSS) than Group 2 (*p* < 0.001). At very high pollen levels, the inter‐group difference was still significant (*p* = 0.02) (Figure 4B and Table 2). In contrast, Group 3 participants took significantly more medication than Group 2 participants, which was most pronounced at low and medium pollen levels (*p* < 0.001) and significant (*p* < 0.05) at all other pollen levels (Figure 4C). When asked: “Did your symptoms impair you in your everyday social activities? If yes, how much so?,” Group 3 participants more often replied with “No, not at all” than Group 2 participants. The inter‐group difference was significant at medium (*p* < 0.001) and at high (*p* < 0.05) pollen levels (Figure 4D).

**TABLE 2:** Results of a two‐way mixed model ANOVA with post hoc Dunnett's test for multiple comparisons.

Multiple comparison	*p*	Summary	Effect size
Group 2 vs. 3: no pollen	0.69	ns	0.7 (medium)
Group 2 vs. 3: low	0.72	ns	0.3 (small)
Group 2 vs. 3: medium	0.0006	***	0.9 (large)
Group 2 vs. 3: high	0.0001	***	1.3 (large)
Group 2 vs. 3: very high	0.02	*	0.7 (medium)
Group	< 0.0001	****	0.2 (small)
Pollen level	< 0.0001	****	0.8 (large)
Group × pollen level	0.23	ns	0.0 (small)

*Note:* The total symptom score (TSS) of the participants of Groups 2 and 3 was compared for different pollen levels. *p < 0.05; ***p < 0.001; ****p < 0.0001.

Abbreviation: ns = nonsignificant.

Figure S6 provides an overview of the symptom diary entries made per day, the symptoms (TSS) versus pollen as a function of calendar date, and the duration of diary participation per participant for the Groups 2 and 3.

### 3.5 Symptom Forecast Based on Baseline‐, Symptom‐ and Environmental Data

A boosted decision tree model (XGBoost) was used to forecast symptoms. Variable distributions of all symptom types that occurred during the study period are shown in Figure S7; class report metrics for all models are reported in Tables S7–S9 in the Supporting Information.

**FIGURE 5:**
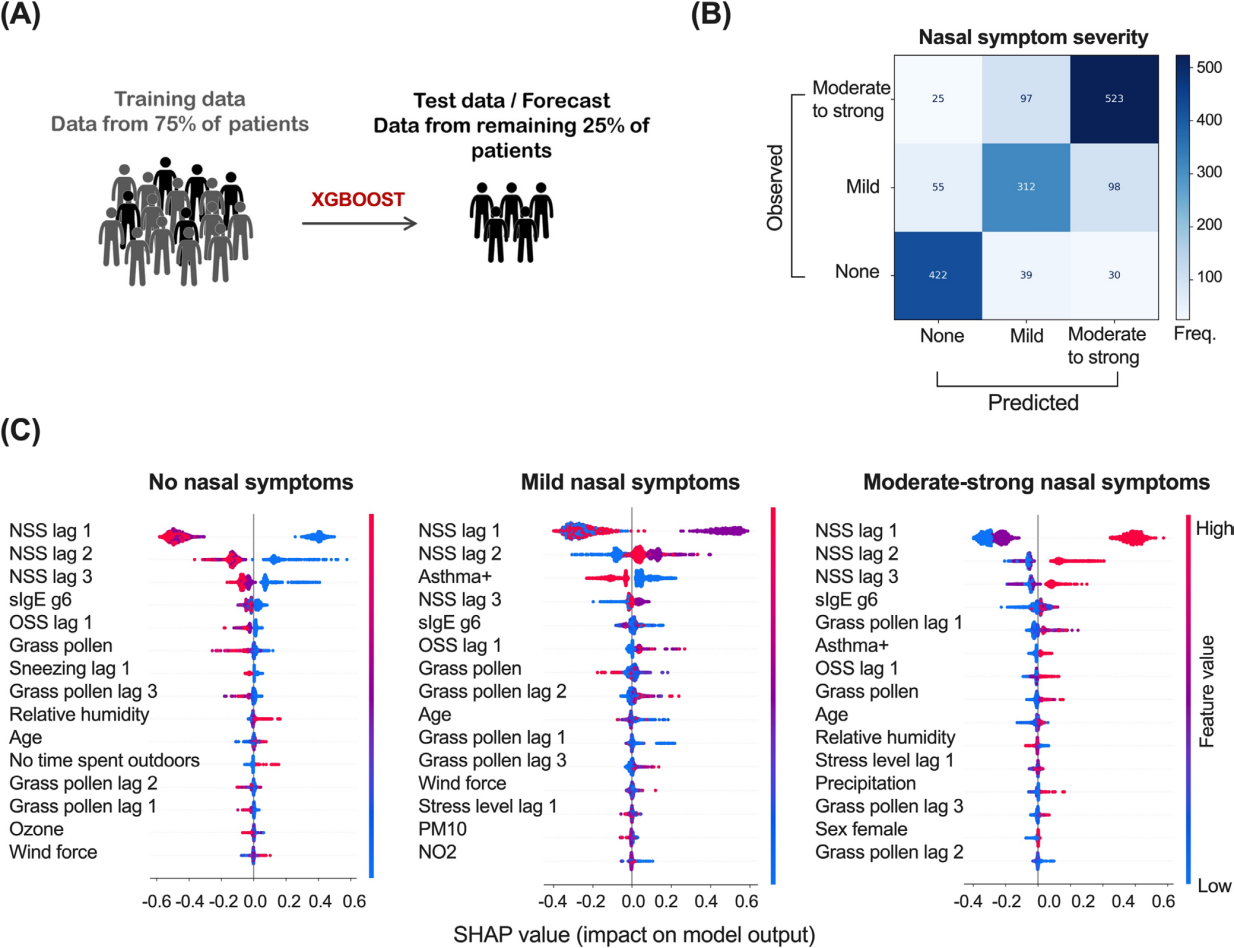
Nasal symptom forecast. (A) A boosted decision tree model (XGBoost) was trained on data from 75% of patients to predict the nasal symptoms of individuals randomly selected from the other 25% of patients. (B) Confusion matrix of predicted (x‐axis) versus observed (y‐axis) nasal symptom levels. Numbers in the squares indicate the frequencies. (C) SHAP value plots with the 15 most important features determining the model output for three symptom severity levels.

To predict organ‐specific symptoms, the model was trained on the data of 75% of patients (4329 diary entries) to forecast the symptom severity category of the remaining 25% of patients (1601 diary entries) (Figure 5A). For the prediction of the nasal symptom category, a macro‐precision of 0.78, a macro‐recall of 0.78, a macro‐F1‐score of 0.78, and an overall accuracy of 0.79 were achieved. The confusion matrix is shown in Figure 5B. The corresponding feature importance for the model's prediction is presented in SHAP summary plots, showing the most important 15 features for each symptom category (Figure 5C).

Ocular symptom prediction was comparably successful (Figure S8), whereas pulmonary symptom prediction failed because too few pulmonary symptoms had been registered (Table S9 and data not shown).

## 4 Discussion

This is the first randomized, controlled study to demonstrate a clinical benefit of an allergy app for SAR patients. A recently published study [18] investigated the effectiveness of an allergy app for the self‐management of SAR and asthma. Our present study adds impact, both in terms of larger sample size and longer time‐series of data. Moreover, our study design enabled us to evaluate different functions of the same app.

The composition of our sample (2/3 women) is rather typical for similar allergy studies in adults [7], in line with previous research suggesting that women have a greater interest in health issues [19] and are more likely than men to utilize health services, such as doctor visits and check‐ups [20]. However, the high intrinsic motivation of the predominantly female participants could represent a potential bias in the cohort. In addition, many of the participants worked in the healthcare sector, which may be associated with a higher level of health awareness and increased willingness to use the app. Finally, all participants received financial compensation, which may also have encouraged high compliance.

Excluding patients with perennial symptoms allows a focused analysis of the effectiveness of the app for seasonal allergies and enables a more accurate assessment of the impact of pollen forecasts on symptom control. In reality, however, many patients suffer from both seasonal and perennial symptoms, so future research should consider the effect of the app on this group of patients in order to increase the generalizability of the results.

Our local pollen forecast proved more accurate than others used in current allergy apps [21]. We saw a highly significant, strong, and positive correlation between the participants' symptoms and the predicted pollen concentrations, with highly symptomatic patients showing the best correlation, illustrating that severe allergy sufferers are more sensitive to pollen exposure.

The diary in itself appeared to provide a benefit on QoL on days with very high pollen level. In contrast, the pollen forecast had a positive effect on QoL even on days with a moderate pollen level. The miniRQLQ proved of limited value due to its short measurement period, therefore, as suggested by similar studies [22, 23], alternative instruments were included a priori. Comparing the QoL‐results with feedback questionnaire answers, our results suggest that QoL is a highly subjective outcome that may be impacted by the intervention alone. Interestingly, only Group 3 participants had rated their symptoms significantly lower with than without app when imagining “a typical grass pollen season,” indicating a possible added value of the pollen forecast in this subjective assessment. The symptom scores show clear differences between the Groups 2 and 3 on days with a moderate to high pollen load. The app with pollen forecast was associated with significantly milder symptoms. A likely reason for the reduced symptoms in Group 3 versus Group 2 is the difference in medication intake: as measured by the app's diary, Group 3 participants, who had timely access to pollen forecast information, took more medication already on days with a low pollen load, thereby possibly reducing their risk to proceed to higher symptoms. Our findings confirm the results of previous observational studies with the MASK app [24, 25, 26]. Apart from differences in medication use, we found no further difference in exposure‐related behavior between the groups. We conclude that the pollen forecast information might lead to unconscious changes in medication intake, independent of the actual pollen level forecasted.

In a previous study, allergic patients had reported higher stress‐related problems [27], and stress could in theory be augmented by an allergy app. If app users are additionally stressed by the prediction of a “high” or “very high” pollen forecast, this could, in principle, worsen their symptoms and therefore cause a nocebo effect. The participants of Group 2, which had used the app without pollen forecast, had higher symptoms than those of Group 3, as measured with the symptom diary, which participants of both groups had filled out daily. This suggests that the app's pollen forecast itself did not worsen the symptoms via raising negative expectations. However, neither of our used measurement instruments (symptom diary, feedback questionnaire) was standardized; therefore, it was not possible to validly assess nocebo effects. This is a limitation of the current study and warrants further investigation in the future using standardized instruments.

Our symptom forecast performed well. Unlike the common approach of using a cross‐validation set to choose between models, we opted to evaluate our model simply by using a test set. This decision was driven by the small sample size and the given class imbalance, which could have otherwise skewed performance metrics for both cross‐validation and test evaluations based on the respective distribution. Of note, our goal was not to validate a model but rather to investigate what is achievable with the given data and, most importantly, to provide insights for future approaches.

A limitation of our symptom forecast is that only data of 83 patients (Groups 2 and 3) could be considered, and when comparing results of the test and train set, overfitting remains still present to a certain extent. This could not be mitigated with the available resources, likely due to (1) limited data availability and (2) the lack of information from the beginning of the pollen season or off‐season periods. Regarding feature importance, it also seems that limited feature availability made predictions overly dependent on previous symptoms, and the partly absence of grass pollen concentration among the top features suggests the model lacked sufficient data to derive pollen as a critical factor. We suggest that including early pollen season data and also off‐season data in future approaches may improve the model's capability to derive the influence of pollen. We further checked whether the symptom forecast would improve for the polysensitized patients by including the other relevant pollen taxa in the dataset. However, in our subsample, we did not find any significant improvement of the model (data not shown). It may still be meaningful to include the entire pollen sensitization profile (and relevant exposure) in future models given a more extensive dataset. Another important limitation is that, at this stage, we cannot tell how our model would perform on a new pollen season.

To achieve a forecast that provides strong clinical value, we chose to predict nasal, ocular, and bronchial symptom severity separately instead of an overall symptom score. An important predictor of nasal symptoms in our forecast was a positive asthma history. Along that line, SAR with concurrent asthma had previously been shown to be associated with lower QoL than SAR without asthma [28].

As reported by others [27], we observed a high correlation between the average symptom score of our cohort and the grass pollen concentration. Thus, while the average symptoms of a population may mostly depend on the grass pollen concentration, individual symptoms, once the season has started, appear to depend largely on patient‐intrinsic factors, for example, sensitization level, severity, and quality of previous symptoms.

Apart from pollen, the most relevant environmental predictors for nasal and ocular symptom severity were wind force, relative humidity, precipitation, PM_10_, NO_2_, and ozone. Ozone is a gaseous irritant known to exacerbate respiratory symptoms [29]. Relative humidity was previously shown to contribute to allergic symptoms in a high‐altitude environment [30]. Our current results are well in line with these findings and stress the relevance of co‐ and multi‐exposures for the development of more severe allergic symptoms.

Taken together, our results add important new insight for developers of allergy apps aiming to provide individualized symptom alerts for pollen allergics. Tailored to the patient's individual symptom and health profile, a symptom forecast may present a useful tool to limit the individual symptom burden [27, 31]. Besides, it could improve treatment behavior by encouraging patients to adjust their therapy early and in a targeted manner. For example, patients could take medication preventively in the event of a foreseeable worsening of their symptoms, leading to better control, and fewer symptom exacerbations. They could also avoid taking medication unnecessarily, which would reduce adverse drug reactions [4]. This is especially important with respect to the widespread self‐medication and ‐management among pollen allergy patients [22, 31, 32] as well as frequent overuse of nasal decongestants [33].

Looking forward, we would like to address the limitations in terms of generalizability. We are already in the process of validating the results presented here in other geographical areas. For example, we have evaluated the same app with local pollen and pollutant forecasts in a rural area from May to September 2023. This study was not limited to grass pollen allergy sufferers but was aimed at population groups living in rural areas (still unpublished).

Further research is needed in this area. Pollen and pollutant forecasting models should be based on regional (real‐time) measurement data in order to respond adequately to short‐term changes. It is crucial that all population groups, including children in particular, benefit from these developments. Finally, allergy apps should be user‐friendly; our symptom diary with 40 questions, for example, would not be practical in everyday life in the long term.

## Author Contributions

Original idea: C.H.; Conceptualization: C.H., A.D., S.G., C.T.‐H.; contribution to the clinical study: S.G., C.H., J.K., P.R., S.S., C.K., G.H.; modeling and statistical analysis: J.K., M.P.P., C.H.; statistical advice: A.D., A.M., J.O.B.; contribution to app workflows: M.R., R.K., M.P.P., J.K.; manuscript draft: C.H., S.G., J.K.

## Conflicts of Interest

The authors declare no conflicts of interest.

## Supporting information


Data S1.

## Data Availability

The data that support the findings of this study are available from the corresponding author upon reasonable request.

## References

[1] J. Bousquet , J. M. Anto , C. Bachert , et al., “Allergic Rhinitis,” Nature Reviews Disease Primers 6, no. 1 (2020): 95. 10.1038/s41572-020-00227-0 33273461

[2] B. J. H. Dierick , T. van der Molen , B. M. J. de Flokstra‐ Blok , et al., “Burden and Socioeconomics of Asthma, Allergic Rhinitis, Atopic Dermatitis and Food Allergy,” Expert Review of Pharmacoeconomics & Outcomes Research 20, no. 5 (2020): 437–453, 10.1080/14737167.2020.1819793. 32902346

[3] B. Sousa‐Pinto , C. Jácome , A. M. Pereira , et al., “Development and Validation of an Electronic Daily Control Score for Asthma (e‐DASTHMA): A Real‐World Direct Patient Data Study,” Lancet Digital Health 5, no. 4 (2023): e227–e238, 10.1016/S2589-7500(23)00020-1. 36872189

[4] M. Ajegbile , J. A. Olaboye , C. C. Maha , G. T. Igwama , and S. Abdul , “The Role of Data‐Driven Initiatives in Enhancing Healthcare Delivery and Patient Retention,” World Journal of Biology Pharmacy and Health Sciences 19 (2024): 234–242.

[5] A. Pizzulli , S. Perna , J. Florack , et al., “The Impact of Telemonitoring on Adherence to Nasal Corticosteroid Treatment in Children With Seasonal Allergic Rhinoconjunctivitis,” Clinical and Experimental Allergy 44, no. 10 (2014): 1246–1254, 10.1111/cea.12386. 25109375

[6] C. Suanno , I. Aloisi , D. Fernández‐González , and S. Del Duca , “Pollen Forecasting and Its Relevance in Pollen Allergen Avoidance,” Environmental Research 200 (2021): 111150, 10.1016/j.envres.2021.111150. 33894233

[7] M. Gökkaya , A. Damialis , T. Nussbaumer , et al., “Defining Biomarkers to Predict Symptoms in Subjects With and Without Allergy Under Natural Pollen Exposure,” Journal of Allergy and Clinical Immunology 146, no. 3 (2020): 583–594.e6. 32272131 10.1016/j.jaci.2020.02.037

[8] K. Karatzas , D. Voukantsis , S. Jäger , et al., “The Patient's Hay‐Fever Diary: Three Years of Results From Germany,” Aerobiologia 30, no. 1 (2014): 1–11.

[9] O. Pfaar , K. Bastl , U. Berger , et al., “Defining Pollen Exposure Times for Clinical Trials of Allergen Immunotherapy for Pollen‐Induced Rhinoconjunctivitis—An EAACI Position Paper,” Allergy 72, no. 5 (2017): 713–722, 10.1111/all.13092. 27874202

[10] L. A. de Weger , K.‐C. Bergmann , A. Rantio‐Lehtimäki , et al., “Impact of Pollen,” in Allergenic Pollen: A Review of the Production, Release, Distribution and Health Impacts, ed. M. Sofiev and K.‐C. Bergmann (Springer Netherlands, 2013), 161–215.

[11] E. F. Juniper , A. K. Thompson , P. J. Ferrie , and J. N. Roberts , “Development and Validation of the Mini Rhinoconjunctivitis Quality of Life Questionnaire,” Clinical and Experimental Allergy 30, no. 1 (2000): 132–140, 10.1046/j.1365-2222.2000.00668.x. 10606940

[12] K. Bastl , M. Kmenta , S. Jäger , K.‐C. Bergmann , and U. Berger , “Development of a Symptom Load Index: Enabling Temporal and Regional Pollen Season Comparisons and Pointing Out the Need for Personalized Pollen Information,” Aerobiologia 30, no. 3 (2014): 269–280, 10.1007/s10453-014-9326-6.

[13] L. Garg , J. Dauwels , A. Earnest , and K. P. Leong , “Tensor‐Based Methods for Handling Missing Data in Quality‐of‐Life Questionnaires,” IEEE Journal of Biomedical and Health Informatics 18, no. 5 (2014): 1571–1580, 10.1109/JBHI.2013.2288803. 24235317

[14] C. Bruch , “Imputation of Missing Values in Survey Data,” 2023, G.‐L. Institute and f.t.S.S.G.‐S. Guidelines, Editors.

[15] Z. Zhang , M. Engardt , M. Stafoggia , and X. Ma , “Improving 3‐Day Deterministic Air Pollution Forecasts Using Machine Learning Algorithms,” Atmospheric Chemistry and Physics 24, no. 2 (2024): 807–851, 10.5194/acp-24-807-2024.

[16] T. Chen and C. Guestrin , “XGBoost: A Scalable Tree Boosting System,” in Proceedings of the 22nd ACM SIGKDD International Conference on Knowledge Discovery and Data Mining (Association for Computing Machinery, 2016), 785–794.

[17] S. M. Lundberg and S.‐I. Lee , “A Unified Approach to Interpreting Model Predictions,” in Proceedings of the 31st International Conference on Neural Information Processing Systems (Curran Associates Inc, 2017), 4768–4777.

[18] V. Landesberger , K. Grenzebach , F. Schreiber , et al., “Conception and Pilot Testing of a Self‐Management Health Application for Patients With Pollen‐Related Allergic Rhinitis and Allergic Asthma‐The APOLLO App,” Scientific Reports 13, no. 1 (2023): 21568, 10.1038/s41598-023-48540-4. 38057347 PMC10700582

[19] S. Winston , “Health Information National Trends Survey (HINTS.Gov),” Medical Reference Services Quarterly 40, no. 2 (2021): 215–223, 10.1080/02763869.2021.1912575. 33970822

[20] P. L. Remington , “The Behavioral Risk Factor Public Health Surveillance System,” American Journal of Preventive Medicine 59, no. 6 (2020): 776–778, 10.1016/j.amepre.2020.09.002. 33220751

[21] K. Bastl , U. Berger , and M. Kmenta , “Evaluation of Pollen Apps Forecasts: The Need for Quality Control in an eHealth Service,” Journal of Medical Internet Research 19, no. 5 (2017): e152. 28483740 10.2196/jmir.7426 PMC5440733

[22] S. Dramburg , S. Perna , M. Di Fraia , et al., “Validation Parameters of Patient‐Generated Data for Digitally Recorded Allergic Rhinitis Symptom and Medication Scores in the @IT.2020 Project: Exploratory Study,” JMIR mHealth and uHealth 10, no. 6 (2022): e31491, 10.2196/31491. 35657659 PMC9206201

[23] A. Bürgler , S. Glick , K. Hartmann , and M. Eeftens , “Rationale and Design of a Panel Study Investigating six Health Effects of Airborne Pollen: The EPOCHAL Study,” Frontiers in Public Health 9 (2021): 689248, 10.3389/fpubh.2021.689248. 34222186 PMC8249754

[24] A. Bédard , X. Basagana , J. M. Anto , et al., “Mobile Technology Offers Novel Insights Into the Control and Treatment of Allergic Rhinitis: The MASK Study,” Journal of Allergy and Clinical Immunology 144, no. 1 (2019): 135–143.e6. 30951790 10.1016/j.jaci.2019.01.053

[25] J. Bousquet , P. Devillier , S. Arnavielhe , et al., “Treatment of Allergic Rhinitis Using Mobile Technology With Real‐World Data: The MASK Observational Pilot Study,” Allergy 73, no. 9 (2018): 1763–1774, 10.1111/all.13406. 29336067

[26] E. Menditto , E. Costa , L. Midão , et al., “Adherence to Treatment in Allergic Rhinitis Using Mobile Technology. The MASK Study,” Clinical and Experimental Allergy 49, no. 4 (2019): 442–460, 10.1111/cea.13333. 30597673

[27] J. D. Silver , K. Spriggs , S. Haberle , C. H. Katelaris , E. J. Newbigin , and E. R. Lampugnani , “Crowd‐Sourced Allergic Rhinitis Symptom Data: The Influence of Environmental and Demographic Factors,” Science of the Total Environment 705 (2020): 135147, 10.1016/j.scitotenv.2019.135147. 31841904

[28] L. Laforest , J. Bousquet , G. Pietri , et al., “Quality of Life During Pollen Season in Patients With Seasonal Allergic Rhinitis With or Without Asthma,” International Archives of Allergy and Immunology 136, no. 3 (2005): 281–286, 10.1159/000083955. 15722638

[29] P. Dabrowiecki , P. Dąbrowiecki , A. Chciałowski , A. Dąbrowiecka , A. Piórkowska , and A. Badyda , “Exposure to Ambient Air Pollutants and Short‐Term Risk for Exacerbations of Allergic Rhinitis: A Time‐Stratified, Case‐Crossover Study in the Three Largest Urban Agglomerations in Poland,” Respiratory Physiology & Neurobiology 315 (2023): 104095, 10.1016/j.resp.2023.104095. 37355057

[30] A. Damialis , F. Häring , M. Gökkaya , et al., “Human Exposure to Airborne Pollen and Relationships With Symptoms and Immune Responses: Indoors Versus Outdoors, Circadian Patterns and Meteorological Effects in Alpine and Urban Environments,” Science of the Total Environment 653 (2019): 190–199, 10.1016/j.scitotenv.2018.10.366. 30408667

[31] B. Sousa‐Pinto , A. Sá‐Sousa , R. J. Vieira , et al., “Behavioural Patterns in Allergic Rhinitis Medication in Europe: A Study Using MASK‐Air() Real‐World Data,” Allergy 77, no. 9 (2022): 2699–2711. 35258105 10.1111/all.15275

[32] A. Muzalyova and J. O. Brunner , “Determinants of the Utilization of Allergy Management Measures Among Hay Fever Sufferers: A Theory‐Based Cross‐Sectional Study,” BMC Public Health 20, no. 1 (2020): 1876, 10.1186/s12889-020-09959-w. 33287774 PMC7720499

[33] E. Mehuys , P. Gevaert , G. Brusselle , et al., “Self‐Medication in Persistent Rhinitis: Overuse of Decongestants in Half of the Patients,” Journal of Allergy and Clinical Immunology. In Practice 2, no. 3 (2014): 313–319, 10.1016/j.jaip.2014.01.009. 24811023

