# Supplementary material for: Clinical Benefits of a Randomized Allergy App Intervention in Grass Pollen Sufferers: A Controlled Trial
Source: Allergy. 2025 Apr 17;80(7):1945–55. doi: 10.1111/all.16558 (PMC12261868; doi:10.1111/all.16558)
Supplement: Supplementary file 1 — Data S1. [file ALL-80-1945-s001.pdf]

Clinical benefits of a randomized allergy app intervention in grass pollen sufferers: a controlled trial

Caroline Holzmann*[1,2], Johannes Karg[1], Matthias Reiger[1,2], Rajiv Kharbal[2], Paola Romano[1,2], Sabrina Scheiwein[3], Claudia Khalfi[1,2], Anna Muzalyova[4], Jens O. Brunner[5,6,7], Gertrud Hammel[1,2], Athanasios Damialis*[8], Claudia Traidl-Hoffmann*[1,2,9], María P. Plaza* [1,2], Stefanie Gilles[#*1,2]

[1] *Institute of Environmental Medicine and Integrative Health, Faculty of Medicine, University Hospital Augsburg, Augsburg, Germany*

[2] *Institute of Environmental Medicine, Helmholtz Munich – German Research Center for Environmental Health, Augsburg, Germany*

[3] *Outpatient clinic for Environmental Medicine, University Hospital Augsburg, Augsburg, Germany*

[4] *Institute for Digital Medicine, University Hospital Augsburg, Augsburg, Germany*

[5] Department of Technology, Management, and Economics, Technical University of Denmark

[6] Next Generation Technology, Region Zealand, Denmark

[7] Health Care Operations/Health Information Management, Faculty of Business and Economics, Faculty of Medicine, University of Augsburg, Augsburg, Germany

[8] *Terrestrial Ecology and Climate Change, Department of Ecology, School of Biology, Faculty of Sciences, Aristotle University of Thessaloniki, Thessaloniki, Greece*

[9] *Christine Kühne - Center for Allergy Research and Education (CK-CARE), Davos, Switzerland*

* *Contributed equally*

[#] *Correspondence to: Stefanie Gilles: stefanie.gilles@med.uni-augsburg.de*

Conflict of interest statement:

All authors declare no conflict of interest.

Sources of funding:

Bavarian State Office for Health and Food Safety

Abstract

Background: Symptom monitoring can improve the adherence to daily medication. However, controlled clinical trials on multi-modular allergy apps and their various functions have been difficult to implement. The objective of this study was to assess the clinical benefit of an allergy app with varying numbers of functions in reducing symptoms and improving quality of life in grass pollen allergic individuals. The secondar objective was to develop a symptom forecast based on patient-derived and environmental data.

Methods: We performed a stratified, controlled intervention study (May-August 2023) with grass pollen allergic participants (N=167) in Augsburg, Germany. Participants were divided into three groups, each receiving the same allergy app, but with increasing numbers of functions. Primary endpoint: rhinitis-related quality of life; Secondary endpoints: symptom scores, relevant behavior, self-reported usefulness of the app, symptom forecast.

Results: Rhinitis-related quality of life was increased in all participants after the intervention, with no statistical inter-group differences. However, participants with access to the full app version including a pollen forecast took more medication and reported lower symptoms and social activity impairment than participants with access to a reduced-function app. Using a XGBoost multiclass classification model we achieved promising results for predicting nasal (accuracy: 0.79) and ocular (accuracy: 0.78) symptom levels and derived feature importance using SHAP as a guidance for future approaches.

Conclusion: Our allergy app with its high-performance pollen forecast, symptom diary and general allergy-related information provides a clinical benefit for allergy sufferers. Reliable symptom forecasts may be created given high quality and high-resolution data.

(248/max. 250 Words)

Keywords: Allergic rhinitis, allergy app, clinical study, pollen forecast, symptom forecast

Supplementary methods

Study design

From 2nd January to 15th July 2023, 225 candidates were screened for eligibility by telephone interview. 58 candidates were excluded due to inadequate sensitization(s), reported perennial symptoms, failure to be re-contacted, or >4 weeks planned absence during the study period. The remaining 167 candidates were included in the study and randomized into the three study arms.

Directly upon randomization, the participants completed the mini-RQLQ via Qualtrics™ and then went on to the intervention phase. The intervention consisted in the daily use of one of three versions of the PollDi app: Group 1 participants used a basic (“placebo”) version of the app with only general information content. Group 2 participants used an intermediate version of the app, consisting in general information content plus symptom diary. Group 3 participants used the full version of the app, consisting in general information content, symptom diary and a pollen and air pollutants forecast. The intervention phase ended for all participants on the 31st of August 2023. Immediately afterwards, the participants filled in the second mini-RQLQ and a feedback questionnaire, which was specifically tailored for the three intervention groups. An overview over the study design is given in **figure S1**. Both questionnaires were completed electronically via Qualtrics™.

The feedback questionnaire can be viewed in the **appendix of the online supplement**.

App’s symptom diary

The symptom diary consisted of 40 questions covering different outcomes, such as general condition (0-10); general stress level (0-10); symptoms due to present or suspected respiratory infection; allergic symptoms: symptom type (ocular-, nasal-, ear-, pharynx/throat-, pulmonary-, gastrointestinal-, neurological-, other symptoms); symptom quality (e. g. nasal congestion, rhinorrhea, sneezing, itch; coded with discrete numerical variables); symptom level on a scale of 0-3 (0 = none; 1 = mild; 2 = moderate; 3 = severe); medication intake (yes/no) and, if yes, type of medication; exposure-relevant behavior (e. g. “Did you sleep with windows open”, followed by multiple choice answers). It also covered selected items related to the quality of life, such as the question “Did your symptoms impair you in your everyday social activities? If yes, how much so?”. A full version of the diary in simple text form is given in the **appendix of the online supplement**.

Pollen data

Airborne grass (Poaceae) pollen was monitored using an automated pollen monitor (Hund BAA500 – "PoMo") located at the Institute for Environmental Medicine and Integrative Health in Augsburg, Germany (48°23′04.15″ N, 10°50′35.95″ E, 4 m a.g.l.). In operation since August 2017, the PoMo extracts pollen using a virtual impactor with a maximum airflow of 6 m³/h, sampling 24 m³ of air daily at three-hour intervals. An image recognition algorithm identifies pollen based on size, shape, and exine and intine characteristics. Results are generated as CSV files, accessible online, with a 4-hour delay and 3-hour resolution. More details about the system are available at https://www.hund.de/en/pollen-monitor.

Weather data

To predict pollen trends, historical pollen concentrations were combined with meteorological parameters. Historical and forecasted hourly meteorological data, including relative humidity, daily mean temperature and precipitation, were obtained from the German Weather Service (DWD) and are publicly available at the Climate Data Center (CDC). For the forecast, which was provided daily, hourly weather data was converted to daily means.

Pollen forecasting

To forecast pollen, an ensemble of models was used that comprised sub-models with varying configurations and weights, aggregating predictions from individual models to minimize the forecasting uncertainty. Seven sub-models were selected for the final ensemble based on individual accuracy metrics (**table S1**); General Linear Model (GLM), Extreme Gradient Boosting (XGBoost), Neural Network timeseries (NNAR), Random Forest (RF), Support Vector Machine (SVM), Hybrid Prophet-XGBoost, and autoregressive moving average (ARIMA).

In a first step, a massive model was tested with 61 models from the Caret package (R studio). To verify the performance of the proposed model, four widely used forecasting accuracy evaluation criteria [1] were chosen to compare the sub-models and the final ensemble model: the RSQ (R^2), the Root Mean Square Error (RMSE), the Mean Absolute Error (MAE), and the Mean Absolute Percentage Error (sMAPE). These metrics had been previously suggested in benchmarking air quality models within the framework of the Air Quality Directive 2008/50/EC (AQD) [2] and they were used to assign the weights for the ensemble model, ensuring that models with better accuracy contributed more significantly to the final predictions.

Three ensemble strategies were tested: ensemble average, simple ensemble median, and weighted ensemble median, which prioritizes higher performing models. The weighted ensemble median demonstrated superior accuracy across all evaluation metrics (**table S2**).

Six years of pollen and weather data (2017-2022) were used for model training, and 2023 was used for validation. Daily pollen concentrations were calculated from 3-hourly raw data and

expressed as a moving average of the previous three days, with the day of the year as a feature. The following formula was used in each model test, taking into account the possible delay in the pollen data and the forecasted weather parameters:

$$\text{Pollen concentration expected} = Mean\,T + RH + Rain + RainLevels + Mov_{Average_{Pollen}} + Lag_{Pollen} + PollenPositive$$

Where *MeanT* refers to the daily mean temperature (ºC), *RH* is the daily mean relative humidity (%), *Rain* is the daily rainfall (mm), *RainLevels* is a categorical variable derived from the rate of precipitation (mm/h) as defined by the World Meteorological Organization, $Mov_{average\ pollen}$ is the moving average of the pollen concentration (grains/m^3) of the three previous days, Lag_{pollen} is the moving average of the pollen concentration (grains/m^3) of the previous day, *PollenPositive* is a factor variable indicating whether on the previous day at least one pollen grain was detected (1) or the pollen concentration was zero (0).

For better comprehension by the app user, the daily pollen concentration obtained from the pollen forecasting model ensemble was transformed and displayed in the app in the categories of *No Pollen, Low, Medium, High* and *Very High* (**table S3**).

Supplementary results

Randomization

All 167 included participants were assigned to one of three study arms (groups) by randomization. The majority (124/167; 85%) of the participants were randomized by the 25th of May 2023 and 15% (25/167) at later dates; the last participant was randomized on the 13th of July 2023 (**figure S2)**.

The randomization criteria were average symptom severity as reported in the screening, age and sex. **Figure S3** shows that the randomization process resulted in three groups of participants (group 1: n = 57; group 2: n = 55; group 3: n = 55), which showed an equal distribution of all selected traits.

More than 80% of group 3 participants considered the pollen forecast as matching their symptoms at least partly

In the feedback questionnaire, we assessed whether and to what extent the pollen forecast matched the symptoms of group 3 participants. In answer to question (Q34): *"Did the app's pollen forecast match the severity of your actual symptoms?"*, none of the men (0%) replied with "very strongly", 29.4% replied with "strongly" and 58.8% with "partly" (overall positive

response rate: 88.2%). On the other hand, 5.9% of men replied with “hardly” and 5.9% with “not at all” (overall negative response rate: 11.8%). Among women, 12.5% replied with “very strongly”, 34.4% with “strongly” and 37.5% with “partly” (overall positive response rate: 84.4%). 12.5% of women replied with “hardly” and 3.1% with “not at all” (overall negative response rate: 15.6%).

Among mildly symptomatic participants (within the 25th average TSS percentile), the responses were 0% “very strongly”, 33.3% “strongly”, 33.3% “partly”, 25% “hardly” and 8.3% “not at all”. Among highly symptomatic participants (within the 75th average TSS percentile), the responses were 16.7% “very strongly”, 25% “strongly”, 41.7% “partly”, 8.3% “hardly” and 8.3% “not at all”.

An overview over the percentages of answers to Q34 is given in **table S4**.

Missing data imputation of miniRQLQ answers did not result in imbalanced samples sizes or randomization target variables

The first miniRQLQ survey (pre-intervention survey) had missing data, which was missing not at random (MNAR). The imputation of missing data with a 3D tensor decomposition method resulted in a new dataset with two complete before-after questionnaires (group 1: n=51; group 2: n=44; group 3: n=51). Missing data imputation resulted in an equal distribution of the randomization target variables age (Kruskal Wallis χ^2 = 1.31, p=0.52), sex (χ^2 = 1.41, p=0.93) and average symptom severity (χ^2 = 0.48, p=0.79) (**figure S4**).

Participants of all groups rated the app useful in terms of quality of life

In addition to the miniRQLQ, we wanted to have an independent tool to measure QoL-related items. Therefore, we asked all participants to complete the following question in the feedback questionnaire (Q9): *“How helpful was the app in terms of quality of life?”*. Two thirds (66.7%) of men answered with “partly” or “strongly”, 2.7% with “very strongly”. Less than a third (30.6%) of men answered with “hardly” or “not at all”. Among women, 68.4% replied with “partly” or “strongly”, 7.1% with “very strongly” and 24.5% gave a negative response (“hardly” or “not at all”). The answer “strongly” was given by 26.5% of group 3 participants, followed by 18.9% of group 1 and 13.3% of group 2 participants. “Partly” was answered by 51% of group 3 participants, followed by 48.9% of group 2 and 47.2% of group 3 participants.

An overview over the percentage of answers per category is provided in **table S5**.

To address the question of potential nocebo effects of app usage, we asked the participants of all three groups whether (Q11) using the app made them think more about their allergy or (Q12), whether the app usage made them increasingly aware of health concerns (**figure S5**). These questions were answered by the majority of participants with “not at all” or “hardly ever”,

regardless of the study group. Inter-group differences in the distribution of answers to Q11 (**figure S5, A**) and Q12 (**figure S5, B**) were not statistically significant (one-way ANOVA).

Symptoms were rated lower with app than symptoms without app

The feedback questionnaire question Q45 *"Imagine a typical grass pollen season. How strong would be your symptoms in such a season (1) without app and (2), with the app?"* was answered by participants of all three groups. The answers were compared between the groups by two-way ANOVA mixed model with post-hoc Dunnett's multiple comparisons test. The inter-group differences were stratified by age and sex, including interaction effects between the variables. **Table S6** shows the results of the statistical analysis.

Figure S6 shows, per day, the number of app diary entries (**Fig. S6, A**), the symptom score (TSS) with respect to the pollen concentration (**Fig. S6, B**), and symptom diary entries made over time of participants of group 2 (**Fig. S6, C**) and group 3 (**Fig. S6, D**).

Symptom forecast based on boosted decision tree modeling

With a boosted decision tree (XGBoost) model we aimed at predicting nasal, ocular and pulmonary symptoms of our study participants. As individuals might have different combinations and degrees of organ-specific symptoms, we attempted to forecast organ-specific symptoms instead of total symptoms. A 3-level symptom severity scale was chosen for this approach based on the skewed frequency distribution for each of the organ-specific symptoms. The frequencies of nasal (**figure S7, A**), ocular (**figure S7, B**) and pulmonary symptoms (**figure S7, C**) that occurred during the study period were highly skewed, with the majority of recorded cases being zero (class 0; no symptoms), followed by the classes of 1 (mild) and 2 (moderate symptoms). Class 3 (severe symptoms) occurred with the lowest frequency for all organ-specific symptoms. The most skewed distribution was observed for pulmonary, the least skewed distribution for nasal symptom frequencies. For the prediction model, we aimed at obtaining less skewed frequencies in the symptom classes. Therefore, we combined the original classes 2 and 3 (moderate and severe) to a single class (moderate to severe). This resulted in a lower degree of frequency skewing, with the frequency distribution of nasal symptoms (**figure S7, D**) showing the lowest degree of skewedness, followed by ocular (**figure S7, E**) and pulmonary (**figure S7, F**) symptoms.

The model was trained on baseline- and symptom data of 75% of patients, then the model was used to predict the ocular symptoms in the test dataset (25% of patients) (**figure S8, A**). The model achieved a macro-precision of 0.78, a macro-recall of 0.75, a macro-f1-score of 0.76 and an overall accuracy of 0.82. **Figure S8, B** shows the confusion matrix of predicted (x-axis) vs. observed (y-axis) ocular symptom levels and **figure S8, C** the respective SHAP value plots of the 15 most important model features.

The class report metrics for the prediction of all symptom-types are shown in the **tables S7-S9**.

Supplementary figures and tables

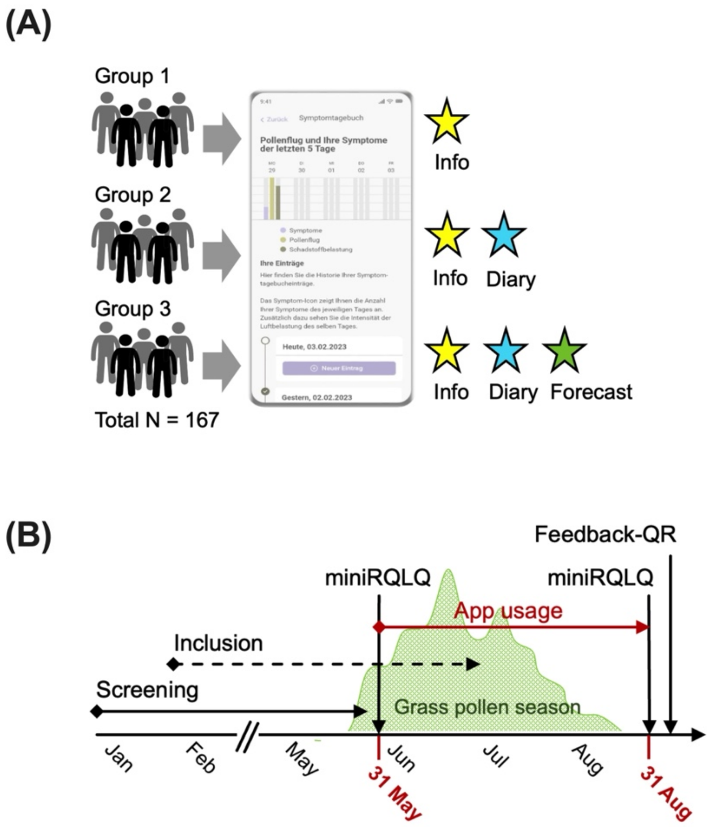

Figure S1: Study groups and interventions. A: Participants were randomly assigned to three groups that received different versions of the PollDi app. Group 1 received a "placebo" app with general information content; group 2 a semi-full app with general information plus symptom diary; group 3 a full app with general information, symptom diary, and pollen- and air-pollutant forecast. **B:** Interventions in the study period.

Model	*MAE*	*MASE*	*sMAPE*	*RMSE*	*RSQ*
GLM	12.61	0.67	56.42	17.11	0.83
XGBoost	14.60	0.77	60.19	21.82	0.77
NNAR	4.58	0.24	37.07	6.26	0.98
RF	21.06	1.11	74.53	26.55	0.64
SVM	13.11	0.69	58.42	18.20	0.83
Hybrid Prophet-XGBoost	15.99	0.85	64.73	21.14	0.82
ARIMA	27.14	1.43	138.01	31.91	0.76

Table S1. Individual accuracy metrics for the seven sub models included in the ensemble model. MAE: Mean absolute error; MASE: Mean absolute scaled error; SMAPE: Symmetric mean absolute percentage error; RMSE: Root mean squared error; RSQ: R^2.

Model	*MAE*	*MASE*	*sMAPE*	*RMSE*	*RSQ*
Ensemble (Mean)	18.49	0.74	75.48	43.39	0.85
Ensemble (Median)	20.34	0.81	72.44	50.67	0.73
Ensemble (Weighted)	18.55	0.74	77.79	42.45	0.86

Table S2. Accuracy metrics for the final ensemble model considered the mean, median or weighted ensemble of the seven sub models. MAE: Mean absolute error; MASE: Mean absolute scaled error; SMAPE: Symmetric mean absolute percentage error; RMSE: Root mean squared error; RSQ: R^2.

**Pollen concentration (grains/m^3)**	**Level**	**Label**
< *3*	0	No Pollen
≥ *3 and < 10*	1	Low
≥ *10 and < 30*	2	Medium
≥ *30 and < 60*	3	High
≥ *60*	4	Very High

Table S3. **Pollen concentration levels based on the pollen forecasting values from the model ensemble.**

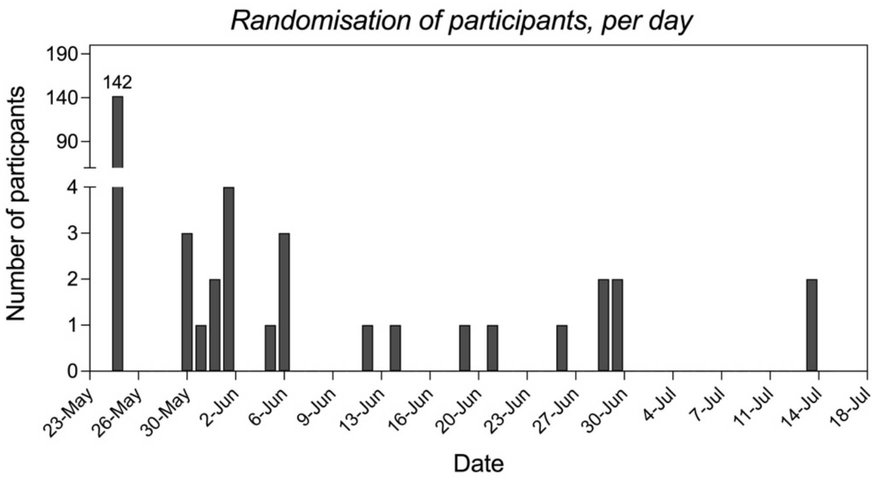

Figure S2: Randomisation of participants as a function of date. The randomization date marked the start of the app-usage. 85% (142/167) participants were randomized on the 25th of May 2023; 15% (25/167) at later dates. The last participant was included on the 13th of July 2023.

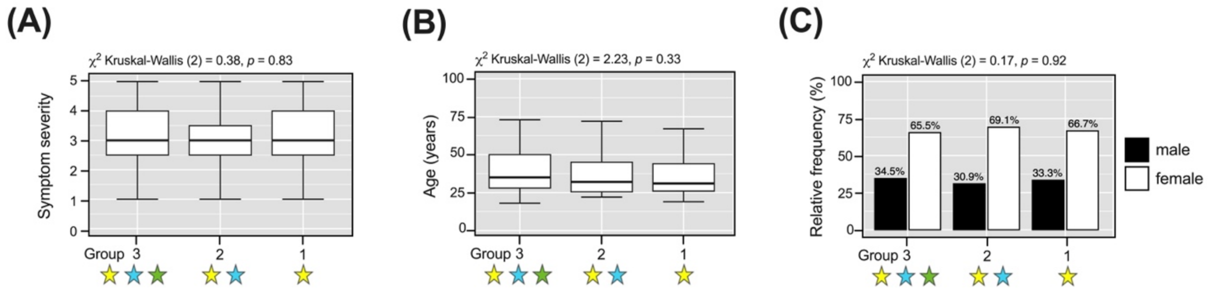

Figure S3: Randomization. Average symptom severity (**A**), age (**B**) and sex (**C**) were equally distributed in all three groups of participants.

		Not at all	Hardly	Partly	Strongly	Very strongly
**Sex**	**male**	5.9	5.9	58.8	29.4	0.0
	**female**	3.1	12.5	37.5	34.4	12.5
**TSS percentile**	**25th**	8.3	25.0	33.3	33.3	0.0
	**75th**	8.3	8.3	41.7	25.0	16.7

Table S4: Answers to feedback questionnaire Q 34*: "Did the app's pollen forecast match the severity of your actual symptoms?"* Overview of the original response options in percent (%). Answers stratified by sex and total symptom score (TSS) percentile. Only participants of group 3, who had received the pollen forecast, were asked to answer this question.

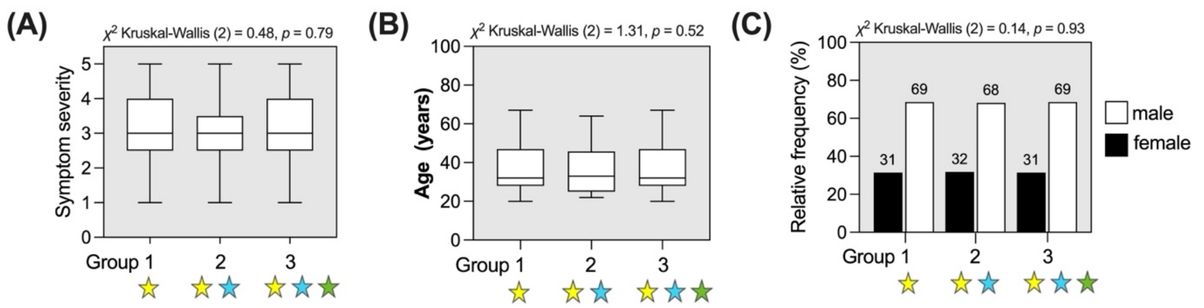

Figure S4. Distribution of the randomization target variables after miniRQLQ missing data imputation. The criteria age (A), sex (B) and average symptom severity, as reported in the screening (C) were equally distributed between the three study arms after imputation of missing data of the pre-intervention miniRQLQ. The sample sizes after imputation were n=51 for group 1, n=44 for group 2 and n=51 or group 3.

		not at all	hardly	partly	strongly	very strongly
**Sex**	**male**	7.7	22.9	41.7	25.0	2.7
	**female**	3.1	21.4	51.0	17.4	7.1
**Group**	**1**	5.7	22.6	47.2	18.9	5.7
	**2**	6.7	26.7	48.9	13.3	6.7
	**3**	2.0	16.3	51.0	26.5	4.1

Table S5: Answers to the QoL-related questionnaire question Q9 of the feedback questionnaire. *Q9: “How helpful was the app in terms of quality of life?”* Overview of the original response options in per cent (%). Answers stratified by sex and study group.

Multiple comparison	p-value	summary	effect size
Without / with app 1	0.59	ns	0.26 (small)
Without / with app 2	0.11	ns	0.38 (small-medium)
Without / with app 3	< 0.0001	****	0.49 (medium)
App usage	0.07	ns	0.21 (small)
Sex	0.71	ns	0.03 (small)
Age	0.25	ns	0.11 (small)
App usage x sex	0.82	ns	0.01 (small)
App usage x age	0.41	ns	0.23 (small)

Table S6. Results of the inter-group comparison of perceived symptom levels with and without app. The perceived symptom severity, as reported in the feedback questionnaire (Q45), was analyzed with a two-way ANOVA mixed model and post-hoc Dunnett’s multiple comparisons. “ns” = non-significant.

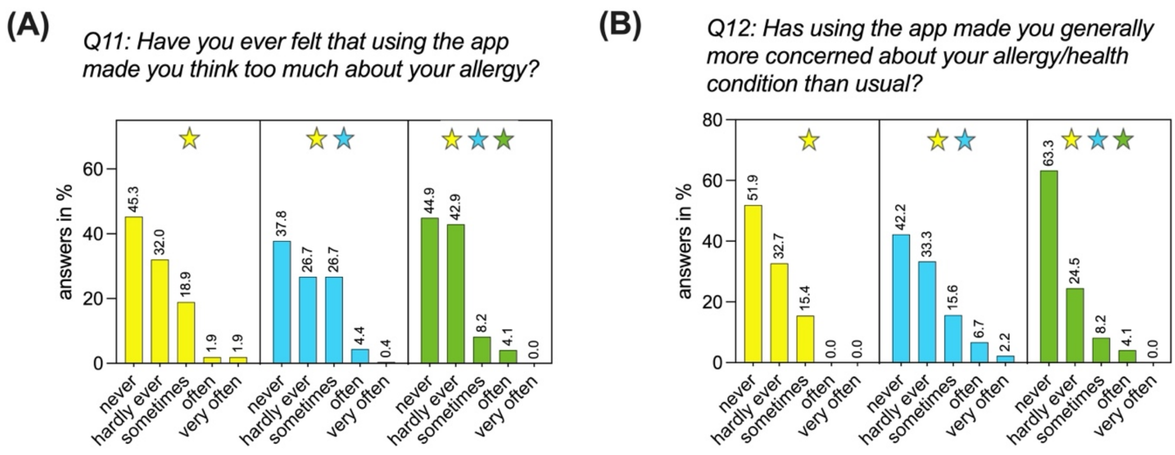

Figure S5: Subjective negative health effects of app usage as reported in the feedback questionnaire. A: Thinking more about allergies. **B**: Increased health concerns. Results are stratified by group.

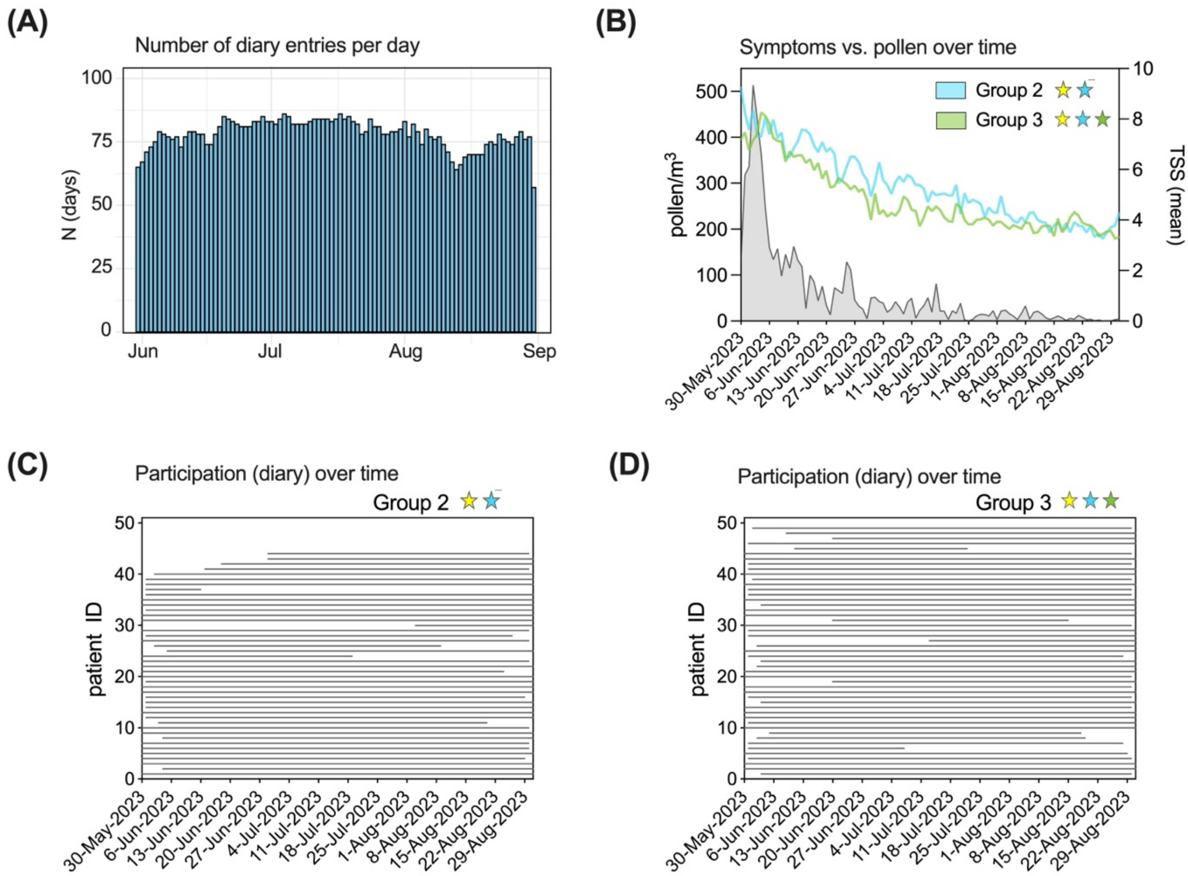

Figure S6: Overview over diary entries and symptoms in groups 2 and 3. A: Number of app diary entries made per day. **B:** Symptom score (TSS) of groups 2 and 3 vs. pollen concentration as a function of calendar date. **C, D:** Symptom diary entries made over time of participants of group 2 (**C**) and group 3 (**D**).

Training data

NSS class	precision	recall	f1-score	support
0	0.89	0.92	0.91	2068
1	0.78	0.73	0.75	1110
2	0.85	0.84	0.84	1151
overall accuracy			0.85	4329
macro avg	0.84	0.83	0.83	4329
weighted avg	0.85	0.85	0.85	4329

Test data

NSS class	precision	recall	f1-score	support
0	0.84	0.86	0.85	491
1	0.70	0.67	0.68	465
2	0.80	0.81	0.81	645
overall accuracy			0.79	1601
macro avg	0.78	0.78	0.78	1601
weighted avg	0.78	0.79	0.78	1601

Table S7. Classification report of the nasal symptom prediction model. NSS: nasal symptom severity. Avg: average.

Training dataset

OSS class	precision	recall	f1-score	support
0	0.94	0.96	0.95	2880
1	0.78	0.75	0.76	710
2	0.91	0.85	0.88	739
accuracy			0.91	4329
macro avg	0.88	0.86	0.87	4329
weighted avg	0.91	0.91	0.91	4329

Test dataset

OSS class	precision	recall	f1-score	support
0	0.89	0.93	0.91	899
1	0.70	0.72	0.71	395

2	0.74	0.62	0.67	307
accuracy			0.82	1601
macro avg	0.78	0.75	0.76	1601
weighted avg	0.81	0.82	0.81	1601

Table S8. Classification report of the ocular symptom prediction model. OSS: ocular symptom severity. Avg: average.

Training dataset				
PSS class	precision	recall	f1-score	support
0	0.97	1.00	0.98	4084
1	0.90	0.57	0.70	207
2	0.94	0.78	0.85	278
accuracy			0.96	4569
macro avg	0.93	0.78	0.84	4569
weighted avg	0.96	0.96	0.96	4569
**Test dataset**				
PSS class	precision	recall	f1-score	support
0	0.91	0.98	0.95	1411
1	0.31	0.14	0.20	35
2	0.80	0.36	0.50	164
accuracy			0.90	1610
macro avg	0.67	0.50	0.55	1610
weighted avg	0.89	0.90	0.89	1610

Table S9. Classification report of the pulmonary symptom prediction model. PSS: pulmonary symptom severity. Avg: average.

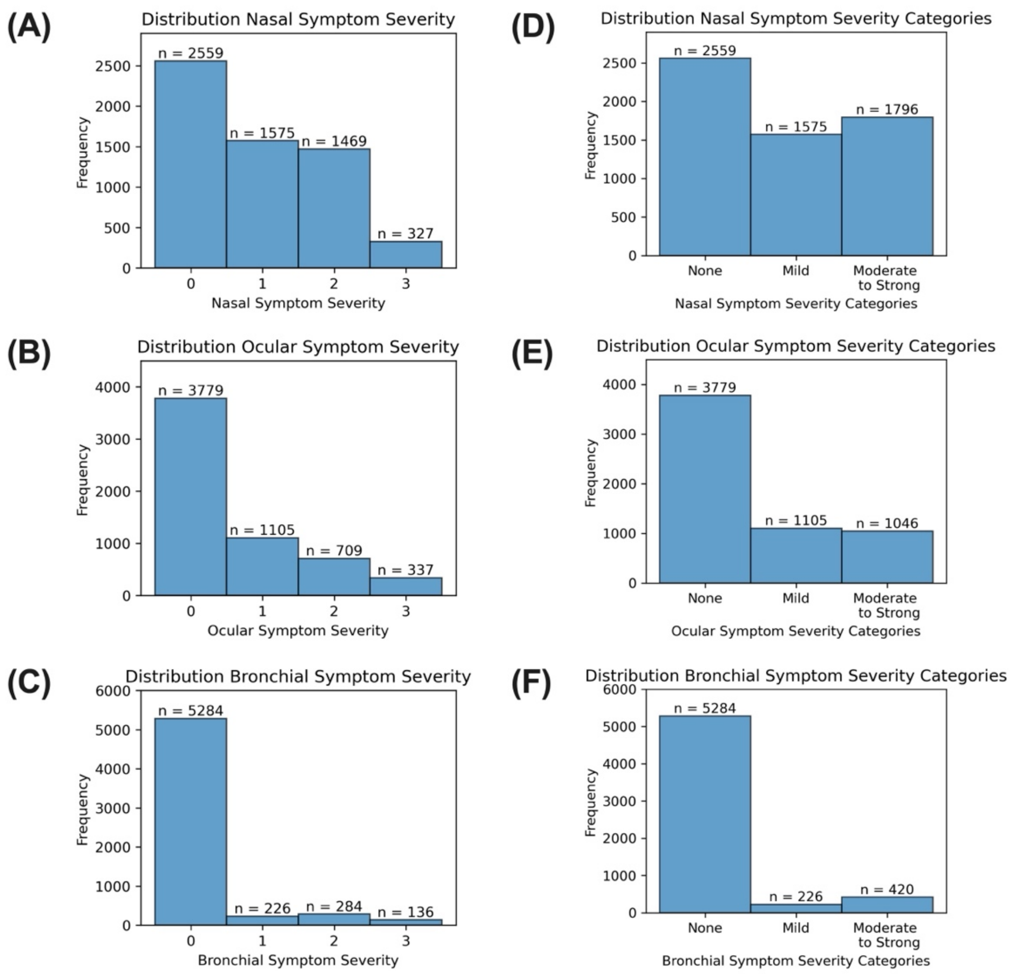

Figure S7: Frequency distribution of symptom severity levels of different scales. A-C: Frequencies of 4-scale nasal (**A**), ocular (**B**) and pulmonary (**C**) symptom severity levels during the study period, with 0 = no symptoms, 1 = mild symptoms, 2 = moderate symptoms, 3 = severe symptoms, and 4 = very severe symptoms. **D-F**: Frequencies of 3-scale nasal (**D**), ocular (**E**) and pulmonary (**F**) symptoms, with 0 = no symptoms, 1 = mild symptoms and 3 = moderate to severe symptoms.

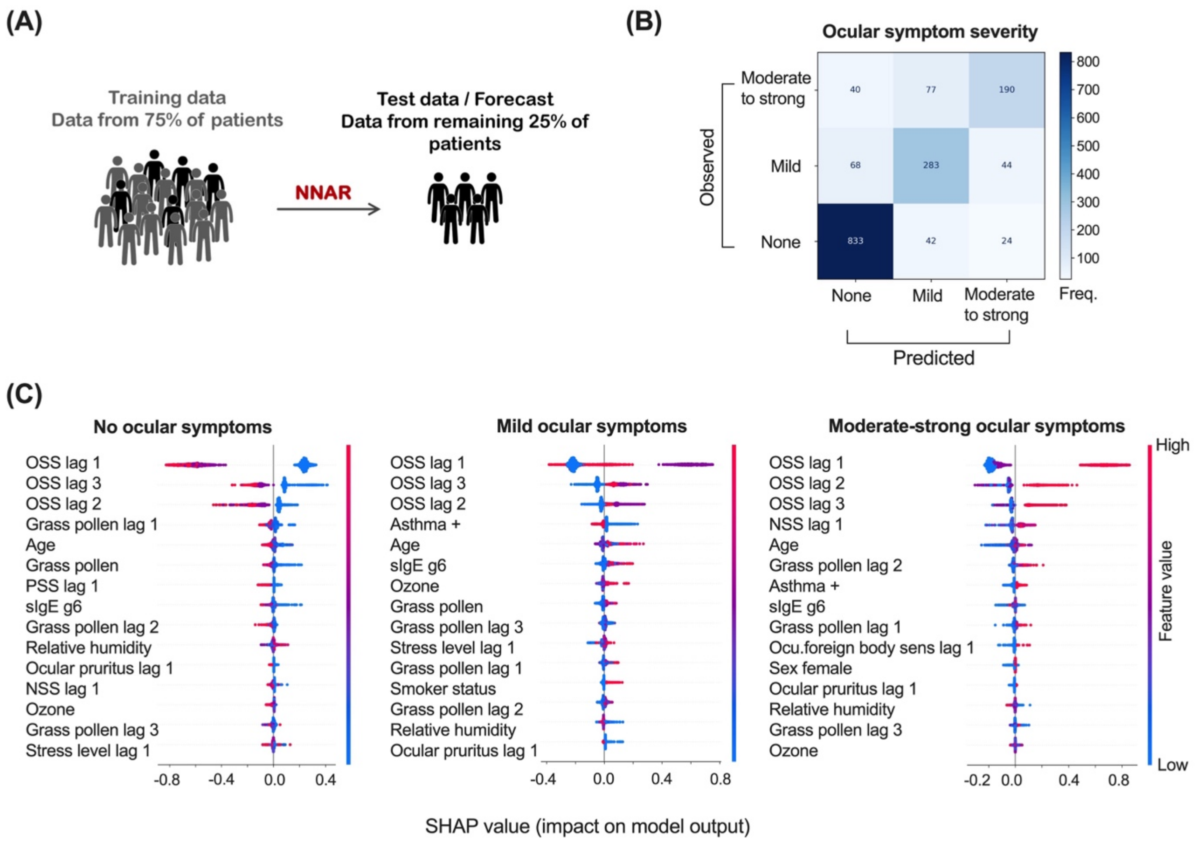

Figure S8: Ocular symptom forecast based on boosted decision tree modeling. A: The model (XGBoost) was trained on data from 75% of patients to predict the ocular symptoms of individuals randomly selected from the other 25% of patients. **B:** Confusion matrix of predicted (x-axis) vs. observed (y-axis) ocular symptom levels. Numbers in the squares indicate the frequencies. **C:** SHAP value plots with the 15 most important features determining the model output for three symptom severity levels.

Appendix

I. Feedback questionnaire

Note: Questions asked to all participants (groups 1, 2 and 3) are in black; questions asked additionally to participants of groups 2 and 3 are highlighted in orange; questions asked in addition to participants of group 3 are highlighted in green.

II. Symptom diary

All questionnaires were originally asked in German and were translated into English by the authors' best knowledge.

1. Zhang, Z., et al., *Improving 3-day deterministic air pollution forecasts using machine learning algorithms.* Atmos. Chem. Phys., 2024. **24**(2): p. 807-851.
2. Janssen, S., Thunis, P., *FAIRMODE Guidance Document on Modelling Quality Objectives and Benchmarking*. 2022.
